# Engineering microbial chemical factories to produce renewable “biomonomers”

**DOI:** 10.3389/fmicb.2012.00313

**Published:** 2012-08-30

**Authors:** Jake Adkins, Shawn Pugh, Rebekah McKenna, David R. Nielsen

**Affiliations:** Chemical Engineering, School for Engineering of Matter, Transport, and Energy, Arizona State UniversityTempe, AZ, USA

**Keywords:** bioplastics, biopolymers, monomers, metabolic engineering

## Abstract

By applying metabolic engineering tools and strategies to engineer synthetic enzyme pathways, the number and diversity of commodity and specialty chemicals that can be derived directly from renewable feedstocks is rapidly and continually expanding. This of course includes a number of monomer building-block chemicals that can be used to produce replacements to many conventional plastic materials. This review aims to highlight numerous recent and important advancements in the microbial production of these so-called “biomonomers.” Relative to naturally-occurring renewable bioplastics, biomonomers offer several important advantages, including improved control over the final polymer structure and purity, the ability to synthesize non-natural copolymers, and allowing products to be excreted from cells which ultimately streamlines downstream recovery and purification. To highlight these features, a handful of biomonomers have been selected as illustrative examples of recent works, including polyamide monomers, styrenic vinyls, hydroxyacids, and diols. Where appropriate, examples of their industrial penetration to date and end-product uses are also highlighted. Novel biomonomers such as these are ultimately paving the way toward new classes of renewable bioplastics that possess a broader diversity of properties than ever before possible.

## Introduction

At present, nearly all conventional plastics are derived from non-renewable natural gas and petroleum resources. Global annual plastics production exceeded 260 million tons in 2009, accounting for roughly 8% of total worldwide oil consumption (Thompson et al., 2009). By just 2010, the total mark had grown to 265 million tons, with major producers China at 23.5% of total production, the European Union at 21.5%, and the rest of Asia contributing another 20% (Plastics Europe, 2011). North American producers, meanwhile, contributed to nearly 20.5% of that mark, consuming approximately 331 million barrels of liquid petroleum gases (LPG) and natural gas liquids (NGL) and nearly 11 billion ft^3^ of natural gas as raw materials in the process (US Energy Information Administration, 2011), in addition to sizable energy demands. All told, in the US, for example, plastics production is responsible for about 4.6% of total consumption of LPG and NGL, 1.5% of total natural gas consumption, and ~1% of total electricity usage. In view of the declining availability of petrochemical feedstocks, the expectation that global plastics production will surpass 297 million tons by 2015 (Global Industry Analysts, 2012), and rising consumer demand for “greener” products, there is increased interest to develop alternative plastics from renewable and sustainable resources.

In contrast to petroleum and natural gas derived plastics, “bioplastics” are produced from the carbon and energy stored in renewable biomass feedstocks. As such, bioplastics offer the promise to reduce dependence on non-renewable oil and natural gas, and can positively impact the carbon cycle by consuming atmospheric CO_2_ (a greenhouse gas) during biomass feedstock cultivation. As the global demand for bioplastics is predicted to triple by 2015 to reach >1 million tons per year to represent a $2.9 billion market (Freedonia Group, 2009), this field is clearly ripe for opportunity and advancements. Whereas technologies exist to convert biomass to plastics through either chemocatalytic processing or by using microbial biocatalysts, the focus here will strictly be on the latter. Together with other recent and complementary reviews (Erickson and NelsonWinters, 2011; Lee et al., 2011a,b, 2012; Curran and Alper, 2012), this article seeks to illustrate how advancements in metabolic and pathway engineering are leading to the development of novel biocatalysts and, in so doing, they are also helping to shape the future of the bioplastics industry.

## Naturally-occurring bioplastics

A number of naturally-occurring biopolymers produced by microorganisms from renewable resources have been found to be useful as bioplastics. The most notable are polyhydroxyalkanoates, or PHAs, which have been extensively reviewed over the years (Braunegg et al., 1998; Chen, 2009; Keshavarz and Roy, 2010). PHAs are biodegradable, linear polyesters composed primarily of monomer subunits of (*R*)-3-hydroxybutyrate (3HB) and are produced as carbon and energy storage molecules by numerous different microbes from both simple and complex biomass feedstocks (note that monomers of 3HB and other β-hydroxyacids will be discussed in detail below). Well-studied examples of bacterial PHA producers include species of *Ralstonia* (Reinecke and Steinbuchel, 2009), *Bacillus* (Singh et al., 2009), and *Pseudomonas* (Rojas-Rosas et al., 2007), as well as phototrophic cyanobacteria (Asada et al., 1999).

Although PHAs are efficiently produced by microbes via naturally evolved biosynthesis routes, expressing the requisite pathway enzymes from various natural producers has also enabled their production at high levels in recombinant *Escherichia coli*, a more tractable host platform (Schubert et al., 1988). All told, bacteria have been engineered to achieve PHA biosynthesis at up to 80% of cell dry weight and at productivities as high as 4 g/L-h from substrates such as glucose (Lee et al., 1999). As crystalline and heat resistant polymers, with glass transition and melting temperatures of around 5°C and 175°C, respectively, PHAs are most well poised as replacements to polyethylene (PE) and polypropylene (PP) (Pei et al., 2011). However, unlike PE and PP, the natural biodegradability PHAs ensures that they will not leave a legacy in landfills at their end of use. Commercial interest in PHAs has been growing, with Metabolix (Cambridge, MA), for example, pursuing their large scale development through their Mirel™ and Mvera™ brands, which collectively are expected to have applications in the production of fibers, films, and coatings, as well as for molded and extruded products.

In spite of numerous advantages, PHA production also suffers from other inherent shortcomings which may ultimately impede their long-term and wide-spread utility. For instance, while their biodegradable nature can help reduce municipal waste, this attribute renders PHAs unsuitable for use in applications requiring long term durability and/or environmental exposure. As all PHAs are thermoplastic polyesters the achievable range of physicochemical and material properties, though diverse, is inherently limited. PHA chain length and purity are difficult to tune and control with high quality assurance in an *in vivo* setting. Lastly, as they are macromolecules, PHAs do not readily diffuse out from cells, but rather accumulate intracellularly as cytosolic inclusion bodies. Thus, since PHAs can only be harvested after the microbial biocatalyst is lysed, prospects for the continuous bioprocessing of PHAs remain unviable.

Another import class of bioplastics that microorganisms naturally produce are those that can be derived from sugars. For example, select strains of *Acetobacter*, most notably *A. xylinum*, are capable of producing high quality cellulose by polymerizing linear chains of β-1,4-glucan (Krystynowicz et al., 2002). Natural cellulose polymers are most attractive due to their inherent biodegradability. Today, most high-grade cellulose is extracted from agricultural sources, for example, fast growing trees or cotton linters. Bacterial cellulose is an attractive alternative, however, because it is free of lignin and hemicelluloses and, with a molecular weight 100-fold lower than plant cellulose, it also possesses greater mechanical strength (Czaja et al., 2006). After synthesizing linear β-1,4-glucan chains and secreting them through pores in their outer membrane, their assembly into microfibril bundles then takes place outside the cell (Ross et al., 1991). Thus, in contrast to PHAs, bacterial cellulose is more amenable to downstream recovery and purification; however, just as PHAs, it still suffers from the same quality assurance and quality control concerns. Nevertheless, bacterial cellulose has found many successful commercial applications. For example, bacterial cellulose is used in Brazil by BioFill Produtos Biotecnologicos in their line of medical films (Biofill®, Bioprocess®, and Gengiflex®) (Hoenich, 2006), whereas Xylos Corp. (Langhorne, PA) also uses it to produce a line of wound care products (XCell®) (Czaja et al., 2006).

## Naturally-occurring biomonomers

Limitations associated with naturally-occurring bioplastics have motivated a recent shift in focus to the alternative production of “biomonomers.” Biomonomers are small molecules (i.e., monomeric subunits) that can undergo *ex situ* chemocatalytic polymerization to produce plastics. This strategy offers several distinct advantages. First, since most biomonomers are often naturally excreted from microorganisms the need for cell collection and lysis is eliminated, greatly reducing operating expenditures and facilitating downstream product recovery. Polymerization of biomonomers in highly controlled chemocatalytic environments leads to bioplastics with finely tuned and predictable properties and at high purities to satisfy quality control specifications. Lastly, the ability to co-polymerize biomonomers with other desired monomers (biologically or otherwise derived) increases the diversity of plastics that can be produced from renewable resources, widening the range of achievable chemistries and material properties.

**Figure 1:**
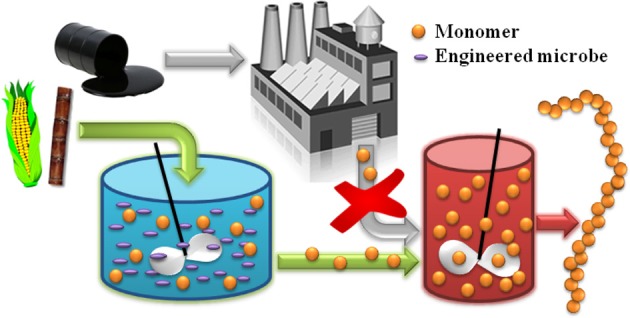
**The alternative production of “drop in compatible” monomer compounds from biomass feedstocks enables the development of new classes of bio-derived polymers by coupling emerging biotechnologies with mature polymer processing technologies**.

In general, there are two classes of biomonomers of industrial interest. The first can be produced from natural metabolites with the aid of a few simple catalytic post-processing steps. For example, the natural fermentation product ethanol can undergo dehydration to ethylene over a solid acid catalyst (Takahara et al., 2005; Hu et al., 2010), before later being polymerized to poly(ethylene) or its co-polymers. Braskem is taking this approach to convert sugarcane-derived ethanol to bioplastics in Brazil (Phillips, 2008). The second class of biomonomers, which will be the central focus of this review, are those metabolites that themselves are directly suitable for polymerization into bioplastics. Wherever possible, the microbial production of “drop in compatible” biomonomers by this approach is advantageous because, as illustrated in Figure 1, it ensures a seamless interface between emerging biotechnologies and the existing polymer industry. This strategy enables decades of technological expertise and existing process infrastructure to be efficiently leveraged, and effectively allows conventional chemical and plastics processing to “pick up where biology leaves off.”

The most prominent example of a naturally-occurring biomonomer is perhaps *L*(+)-lactic acid, whose polymerization leads to the polyester poly(lactic acid), or PLA. *L*(+)-Lactic acid is a natural fermentation product of numerous microbes, of which prominent examples include lactic acid bacteria (including numerous *Lactobacilli* sp.) and filamentous fungi (Kosakai et al., 1997; Zhou et al., 1999). As microbial lactic acid production has been extensively reviewed in the past (Wee et al., 2006; Reddy et al., 2008), it will not be addressed in detail here. It is worth noting, however, that *E. coli* been engineered to over-produce both *L*(+)- (Zhou et al., 1999) and *D*(−)- (Zhou et al., 2003; Mazumdar et al., 2010) stereroisomers of lactic acid as optically pure products. This is important because lactic acid stereochemistry greatly controls relevant physical properties of PLA including, for example, crystallinity and in turn melting point (Lunt, 1998; Sodergard and Stolt, 2002). In a related and interesting study, Lee and coworkers recently engineered *E. coli* for the direct *in vivo* production of PLA by modifying the substrate specificity of PHA synthase 1 of a *Pseudomonas* sp. and promoting the production of lactyl-CoA, the monomer precursor, by introducing an evolved propionate CoA-transferase from *Clostridium propionicum* (Jung et al., 2010; Yang et al., 2010). However, just as PHA is confined within cells, so too is *in vivo* produced PLA. Thus, it is not yet clear how easily or well this specific approach will translate to the commercial scale. Today, NatureWorks LLC (Minnetonka, MN) already produces > 20 different grades of their Ingeo™ PLA bioplastics with structures that range from amorphous to crystalline and uses spanning from films to foams. Like PHAs, PLA continues to be a desirable target due in large part to its biodegradability.

A second naturally-occurring biomonomer that has been extensively investigated to date is succinic acid. This 4-carbon diacid is a natural fermentation product of numerous bacteria, including *Actinobacillus succinogenes*, *Anaerobiospirillum succiniciproducens*, and *Mannheimia succiniciproducens*, which have been reviewed elsewhere (Song and Lee, 2006). Moreover, *E. coli's metabolism* has again also been extensively engineered to over-produce this minor fermentation product to near theoretical yields (Thakker et al., 2012). Succinic acid is particularly useful for producing both polyesters and polyamides, and the first commercial bioplastics derived from this biomonomer will likely be polyamide-5,4 (Qian et al., 2009) and/or poly(butylene*-co-*succinate) (PBS) (Bechthold et al., 2008), a biodegradable thermoplastic suitable for extrusion, injection molding, thermoforming, and film blowing (Xu and Guo, 2010). As microbial succinic acid production is now becoming a mature technology, several producers, including both Myriant (Quincy, MA) and BioAmber (Plymouth, MN), are now developing commercial scale fermentation processes. BioAmber, for example, currently operates a 3000 ton capacity plant in France, with plans to commission a 17,000 ton facility in Canada in 2013.

## Engineering novel biomonomers

Despite the potential advantages of using biomonomers to produce renewable plastics, a limited pool of useful naturally-occurring metabolites ultimately constricts the diversity of bioplastics that can be produced by this approach. However, by applying metabolic engineering tools and strategies, *de novo* metabolic pathway engineering provides the potential to produce non-natural biomonomers, and novel bioplastics, that were never before possible.

**Figure 2:**
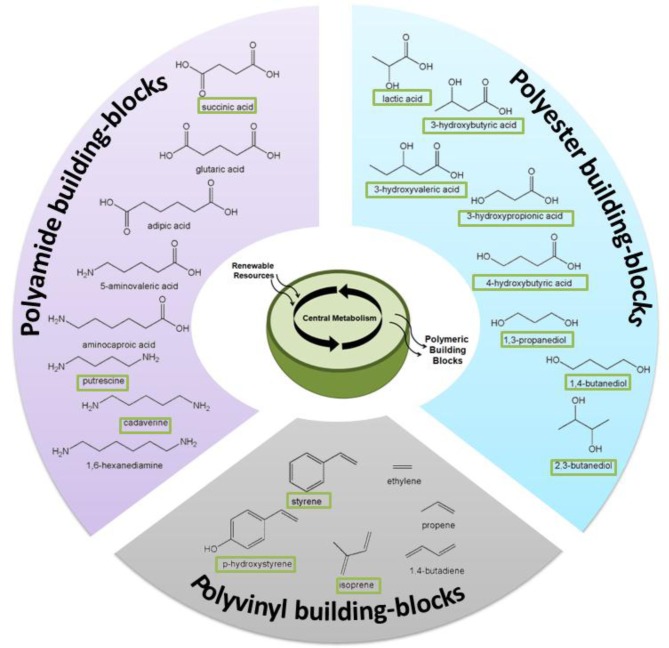
**Numerous biomonomers can now be produced as a result of metabolic and pathway engineering; examples discussed in this review are depicted here**. “Boxed” names signify molecules that have been produced directly from renewable resources, whereas others have been produced via a hybrid, biocatalytic-chemocatalytic approach.

Through the rational re-engineering of microbial metabolism “microbial chemical factories” can be constructed to convert biomass feedstocks into chemical products of high value and/or utility as greener and more sustainable alternatives to petrochemicals (Carothers et al., 2009; Martin et al., 2009). This approach has already led to significant and recent improvements to the production of renewable fuels (Atsumi et al., 2008; Atsumi and Liao, 2008; Peralta-Yahya and Keasling, 2010; Schirmer et al., 2010; Steen et al., 2010; Mendez-Perez et al., 2011; Rude et al., 2011), chemicals (Wierckx et al., 2005; Qian et al., 2009; Kind et al., 2010a; Schirmer et al., 2010; Whited et al., 2010), and active pharmaceutical ingredients (Ro et al., 2006; Leonard et al., 2009; Tsuruta et al., 2009; Campbell and Vederas, 2010). As illustrated by several examples in Figure 2, these strategies now offer the same potential to enhance production of novel biomonomers and, as the following selected examples will illustrate, significant progress is already being made in this regard.

## Polyamide biomonomers

**Figure 3:**
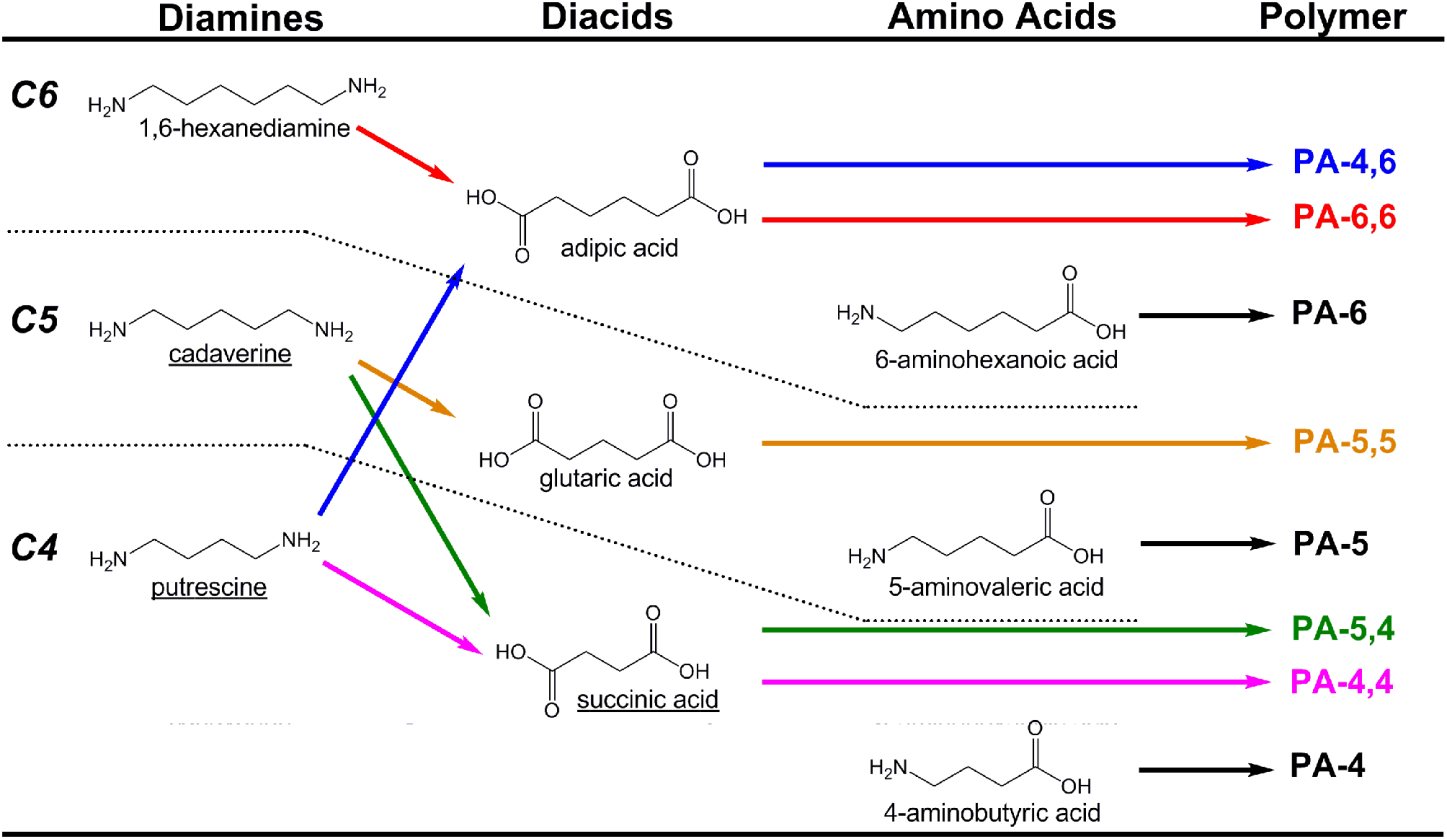
**Examples of polyamides (PAs) produced from combinations of C4–C6 diamines, diacids, and amino acids**. To date, molecules with names underlined have been produced microbially from renewable resources with the aid of metabolic engineering.

Polyamides (PAs; also known as nylons) are a class of plastics that balance mechanical strength and durability with chemical resistance, and which find extensive use as textiles and mechanical parts. PAs are formed either as homopolymers of amino acids or as co-polymers via condensation of diamines with diacids. The most common commercial PAs are PA-6 (a homopolymer of 6-aminohexanoic acid) and PA-6,6 (a copolymer of adipic acid and 1,6-hexanediamine) which account for more than 85–90% of the global market, which will approach 6.6 million tons by 2015 (Nexant Chem Systems, 2009). As carbon chain length between amide bonds (typically 2–12) strongly influences material properties, a large diversity of PAs can be generated from a limited monomer pool by a combinatorial approach. Here, however, we specifically focus on recent progress and opportunities related to the microbial production of 4- through 6-carbon linear diamines, amino acids, and diacids (Figure 3).

Putrescine, the 4-carbon diamine counterpart to succinic acid, occurs as an amino acid biodegradation product, however no natural over-producers have been isolated to date. Putrescine can be produced by *E. coli* by two different pathways that stem from either L-arginine (through agmatine by SpeAB) or L-ornithine (from L-glutamate by SpeC and SpeF) (Tabor and Tabor, 1985; Igarashi and Kashiwagi, 2000). Several researchers have taken to rationally engineering putrescine over-producing microbes including Qian et al. who, for example, developed a putrescine over-producing *E. coli* strain by increasing metabolite flux toward L-ornithine (Qian et al., 2009). This was achieved through both promoter replacements and targeted deletions to overcome allosteric and transcriptional regulation of the L-ornithine biosynthesis pathway. Transcriptional repression by L-arginine upon the native promoters P_argCBH_, P_argD_, and P_argE_ was alleviated by their replacement with the strong promoter P_trc_. Meanwhile, P_speC_ and P_sepFpotE_ were also replaced with P_trc_ to relieve feed-back regulation by putrescine and to increase putrescine export through the *potE* exporter, respectively. Multiple competing pathways were deleted and the resultant *E. coli* strain produced 24.2 g/L putrescine from glucose in fed-batch cultures (Qian et al., 2009). *Corynebacterium glutamicum* has also been used for engineering putrescine over-production by both the L-ornithine and L-arginine pathways, however, the L-arginine pathway was ~40-fold less effective, most likely due to the inhibitory effects of the urea byproduct (Schneider and Wendisch, 2010). With the L-ornithine pathway, expression of L-ornithine decarboxylase from *E. coli* resulted in putrescine titers of up to 6 g/L (Schneider and Wendisch, 2010). Thus, the relative utility of the L-ornithine-derived pathway is conserved between both host platforms investigated. DSM Engineering Plastics (The Netherlands) currently uses putrescine derived from castor oil to produce PA-4,6 and PA-4,10, marketed as Stanyl™ and EcoPaXX™, respectively. Microbial putrescine production from simple sugars, however, would add economic and sustainability benefits.

**Figure 4:**
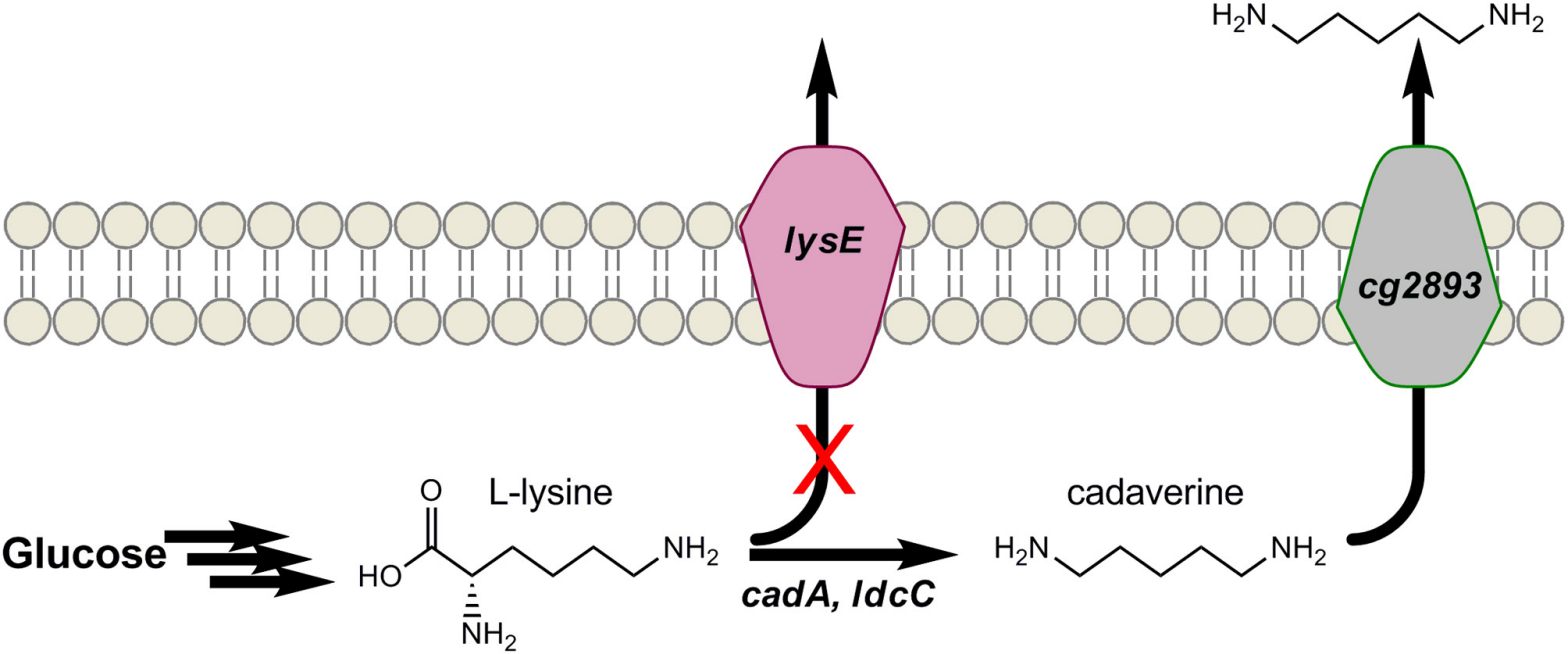
**Engineering cadaverine production in Corynebacterium glutamicum by (1) over-expressing L-lysine decarboxylase (*cadA*), (2) disrupting L-lysine the exporter (*lysE*), and (3) over-expressing an identified cadaverine exporter (*cg2893*)**.

Similar to putrescine, the 5-carbon diamine cadaverine also naturally occurs as an amino acid decarboxylation product, and only at low levels. In this case, it is the decarboxylation of L-lysine that forms cadaverine. PAs derived from cadaverine possess such desirable properties as high melting points and low water absorption (Kind and Wittmann, 2011). In addition, PA-5,4 and PA-5,10 have been proposed as bio-based alternatives to several petroleum-derived PAs (Kind and Wittmann, 2011; Qian et al., 2011). Mimitsuka et al. were first to engineer a microbe for cadaverine over-production from glucose by inserting the acid inducible lysine decarboxylase (encoded by *cadA*) of *E. coli* into the genome of a L-lysine over-producing strain of *C. glutamicum*, as seen in Figure 4 (Mimitsuka et al., 2007). The same basic strategy has since been implemented in both *E. coli* and *C. glutamicum* which, through additional engineering, have produced cadaverine up to 9.6 g/L in *E. coli* and over 10 g/L in *C. glutamicum* on renewable substrates (Mimitsuka et al., 2007; Tateno et al., 2009; Kind et al., 2010a; Qian et al., 2011). For example, to further enhance production in *E. coli*, Qian et al. subsequently deleted the native enzymes spermacetyl transferase, spermidine synthase, cadaverine oxidase, and putrescine aminotransferase (encoded by *speG*, *speE*, *ygjG*, and *puuA*, respectively) which use cadaverine as substrate (Qian et al., 2011). In *C. glutamicum*, Kind et al. recently showed *ldcC* of *E. coli* functions better than *cadA* as a result of differences in pH optima (Kind et al., 2010a), and codon-optimization further aided its function. To increase the availability of L-lysine, *lysE* was deleted to eliminate losses due to lysine export from *C. glutamicum* (Figure 4) (Kind et al., 2010b). Genome-wide transcript analysis identified a putative permease (encoded by *cg2893*) that was up-regulated in the presence of cadaverine. While deletion of *cg2893* reduced cadaverine production by 90%, its over-expression using the strong promoter P_sod_ improved cadaverine yields by 20% (Kind et al., 2011). These results show that if cadaverine can be effectively exported from cells, the negative impacts of allosteric product feed-back inhibition on engineered pathways can be reduced. Most importantly, strategies analogous to those developed here may be of broader utility to the production of other biomonomers and biochemicals.

PA homopolymers are produced from amino acid monomers, a 4-carbon example of which is 4- or γ-aminobutyrate (GABA). GABA is naturally produced by many organisms from L-glutamate via glutamate decarboxylase. Whereas a microbe that produces GABA directly from glucose has yet to be reported, GABA production from exogenously fed L-glutamate has been investigated in strains engineered to over-express heterologous glutamate decarboxylase. For example, up to 5.5 g/L GABA was produced from 10 g/L of glutamate by *E. coli* expressing glutamate decarboxylase (encoded by *gadB*) and the GABA/Glutamate antiporter (encoded by *gadC*) (Le Vo et al., 2012). Additionally, the GABA aminotransferase (encoded by *gabT*) was deleted to prevent endogenous GABA degradation. Future engineering of L-glutamate over-producing hosts will no doubt lead to strains that can achieve high-level GABA production directly from glucose.

As illustrated in Figure 3, the bio-production of several other useful PA monomers has yet to be realized. Like cadaverine, the 5-carbon diacid glutaric acid is known to be a natural L-lysine degradation product. Numerous *Pseudomonas* sp., for example, are known to degrade L-lysine through glutaric acid via 5-aminovaleric acid (AMV) in the AMV pathway (Revelles et al., 2007). Thus, if the AMV pathway can be functionally reconstructed in a L-lysine over-producing host, renewable routes to PA-5 and PA-5,5 would be possible. While PA-5 has not achieved commercial realization, it is known to possess properties close to PA-4,6 and could serve as a suitable substitute (Bermudez et al., 2000). Meanwhile, the microbial production of three of the most important conventional PA monomers, namely 6-aminohexanoic acid (precursor to PA-6), adipic acid, and 1,6-hexanediamine (both precursors to PA-6,6) has yet to be demonstrated. The closest demonstration was of adipic acid production, from Niu et al. who engineered *E. coli* to produce *cis,cis*-muconic acid from glucose at final concentrations of 36 g/L (Niu et al., 2002). *cis,cis*-Muconic acid can be hydrogenated over a palladium catalyst at high pressures to yield adipic acid. Certainly, however, a “one pot” biosynthetic approach would be preferred.

## Styrenic vinyls

**Figure 5:**
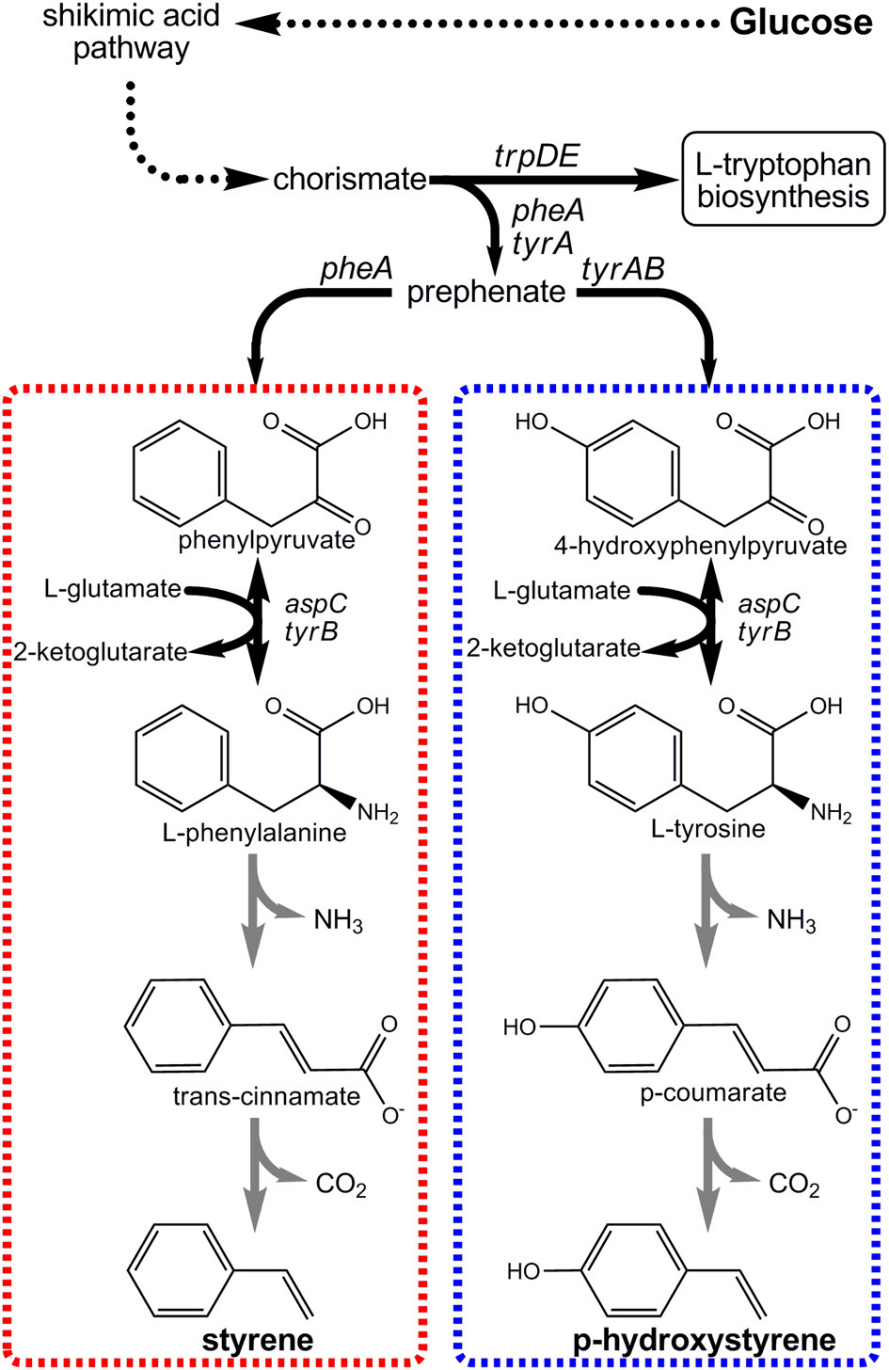
**Novel pathways engineered for the production of p-hydroxystyrene and styrene from renewable glucose via aromatic amino acid precursors**.

Styrenic vinyls are a family of polymers and co-polymers that use styrene or substituted styrene as a key monomer building-block. Today, nearly 60% of styrene's global annual production (about six million tons in the US manufacturers and 24 million tons globally), for example, supports plastics production (Sri, 2010). Examples include polystyrene (PS), acrylonitrile-butadiene-styrene (ABS), styrene-acrylonitrile (SAN), and styrene butadiene rubber (SBR). Low density PS foams, for instance, are widely used in packaging and as thermal insulators (Thorball, 1967), while the “rubbery” characteristics of SBR make it a natural rubber alternative and the major constituent of tires and many gaskets. Today, all styrenic plastics are produced using petroleum-derived monomers. For example, styrene is produced via ethylbenzene dehydrogenation (Wu et al., 1981). This reaction, however, demands over 3 metric tons of steam per ton of styrene produced, rendering styrene as one of the most energy-intensive commodity petrochemicals (US Department of Energy, 2002). The recent engineering of novel enzymatic routes to both p-hydroxystyrene (pHS) and styrene and from glucose (Figure 5), however, may soon enable their renewable production.

The first styrenic biomonomer to be synthesized from renewable resources was pHS (Qi et al., 2007). Its innate optical properties make pHS useful for producing photoresist polymers used in semiconductor manufacturing, for example (Pawlowski et al., 1991). As seen in Figure 5, the engineered pHS biosynthesis pathway stemmed from L-tyrosine as its immediate endogenous precursor. Only two enzymatic steps were then required to convert L-tyrosine to pHS. First, the bi-functional phenylalanine/tyrosine ammonia lyase (PAL/TAL) of the yeast *Rhodotorula glutinis* catalyzed p-coumaric acid formation. Then, p-coumaric acid was subsequently decarboxylated by phenylacrylate decarboxylase of either *Bacillus subtilis* (encoded by *pdc*) or *Lactobacillus plantarum* (encoded by *padC*). Initial *E. coli* strains expressing the pHS pathway achieved final titers as high as only 400 mg/L. Accumulation beyond this mark was severely limited as a result of pHS toxicity imposed on the *E. coli* host (Qi et al., 2007). To overcome toxicity limitations, the well-known aromatic-tolerant *Pseudomonas putida* S12 was next investigated as a pHS production platform. Utilizing two strains derived from *P. putida* S12 to overproduce precursor L-tyrosine as hosts, the pHS pathway was introduced. Due to the innate ability of *P. putida* S12 to degrade numerous aromatic solvents, multiple competing pathway genes were also targeted for deletion. These included both *smo*, which encodes styrene monooxygenase, and *fcs*, the first gene in the p-coumaric acid degradation pathway. All told, engineered *P. putida* S12 strains were able to reach and tolerate pHS titers as high as 545 mg/L, nearly a 50% improvement.

More recently, our own group engineered a novel pathway to enable *E. coli* to produce styrene directly from glucose (McKenna and Nielsen, 2011). The styrene biosynthetic pathway is analogous to the pHS pathway (Figure 5), except in that it stems from L-phenylalanine as its endogenous precursor and as such requires different pathway enzyme “parts.” In this case, L-phenylalanine ammonia lyase (PAL) first converts L-phenylalanine to trans-cinnamic acid which is then decarboxylated to styrene. Upon screening numerous enzyme candidates, it was ultimately found that PAL2 from *Arabidopsis thaliana* and FDC1 from *Saccharomyces cerevisiae* were most effective for these steps, respectively. By over-expressing PAL2 and FDC1 in a L-phenylalanine over-producing *E. coli* host, styrene titers as high as 264 mg/L were achieved. As with pHS, styrene titers too approached *E. coli*'s toxicity limit (~300 mg/L) and must similarly be overcome if economical styrene biomonomer production is to be achieved.

## Hydroxyacids

In recent years, much attention has been given to the bioproduction of the enantiomerically pure hydroxyacids. Not only is this class of molecules useful as precursors for the production of pharmaceuticals, vitamins, antibiotics, and flavor compounds (Tseng et al., 2009), but hydroxyacids can also serve as biomonomers to derive other renewable polyesters. Hydroxyacids of interest in bioplastics include terminal hydroxyacids such as 3-hydroxypropionate (3HP), as well as the β-hydroxyacids 3-hydroxybutyrate (3HB) and 3-hydroxyvalerate (3HV). Each of these hydroxyacid monomers have been produced microbially from renewable resources via pathway engineering.

The shortest of the hydroxyacid monomers is 3HP. 3HP was previously named one of the 12 “Top Value Added Chemicals from Biomass” by the US Department of Energy (2004). In addition to serving as polyester monomer, 3HP can also be used to produce several other specialty and commodity chemicals through additional chemocatalytic processing (Rathnasingh et al., 2009), including the conventional monomers acrylamide, propiolactone, malonic acid, and 1,3-propanediol. The most notable of which, however, is acrylic acid produced by the dehydration of 3HP. Acrylic acid and its esters are used in polymeric flocculants, dispersants, coatings, paints, and adhesives (Straathof et al., 2005) and represent a $10 billion global market. Several companies are now pursuing the commercial development of 3HP-derived acrylic acid, including, for example, OPX Biotechnologies (Boulder, CO).

While several distinct enzymatic routes have been proposed for microbial 3HP production (Jiang et al., 2009), two have demonstrated the greatest promise to date. The first was a two-step pathway consisting of an adenosylcobalamin dependent glycerol dehydratase to catalyze the dehydration of glycerol to 3-hydroxypropionaldehyde (3HPA), and an aldehyde dehydrogenase to oxidize 3HPA to 3HP (Suthers and Cameron, 2000). Using this pathway, Rathnasingh and coworkers engineered *E. coli* to produce 38.7 g/L 3HP in a fed-batch bioreactor with glycerol as substrate. In their strain, glycerol dehydratase (DhaB) from *Klebsiella pneumoniae* DSM 2026 and α-ketoglutaric semialdehyde dehydrogenase (KGSADH) from *Azospirillum brasilense* were selected (Rathnasingh et al., 2009). A key shortcoming of this approach, however, was the uneconomical requirement for exogenous coenzyme-B12 supplementation. To address this limitation, *E. coli* was later engineered to produce 3HP from glucose in a coenzyme-B12-independent manner. This second 3HP pathway consisted of over-expressing the acetyl-CoA carboxylase and biotinilase genes of *E. coli* K-12 (*accADBCb*) to convert endogenously produced acetyl-CoA to malonyl-CoA, and expressing the NADPH-dependent malonyl-CoA reductase gene (*mcr*) of *Chloroflexus aurantiacus* DSM 635 to reduce malonyl-CoA to 3HP (Rathnasingh et al., 2012). This second pathway, however, initially suffered from inherent NADPH limitations under aerobic conditions. This was ultimately addressed by co-expressing nicotinamide nucleotide transhydrogenase (*pntAB* from *E. coli*) to convert NADH (the more abundant reducing equivalent) to NADPH, leading to 3HP titers of up to 0.19 g/L (Rathnasingh et al., 2012).

Although both 3HP pathways possess their own unique limitations which hinder their industrial potential, these pathways represent platforms to which subsequent improvements can now be made via continued strain development. For example, the B12-dependent pathway from glycerol may be well-suited for use in natural organisms which are employed for the industrial production of coenzyme-B12, such as *Propionibacterium shermanii* or *Pseudomonas denitrificans*, for example (Martens et al., 2002).

**Figure 6:**
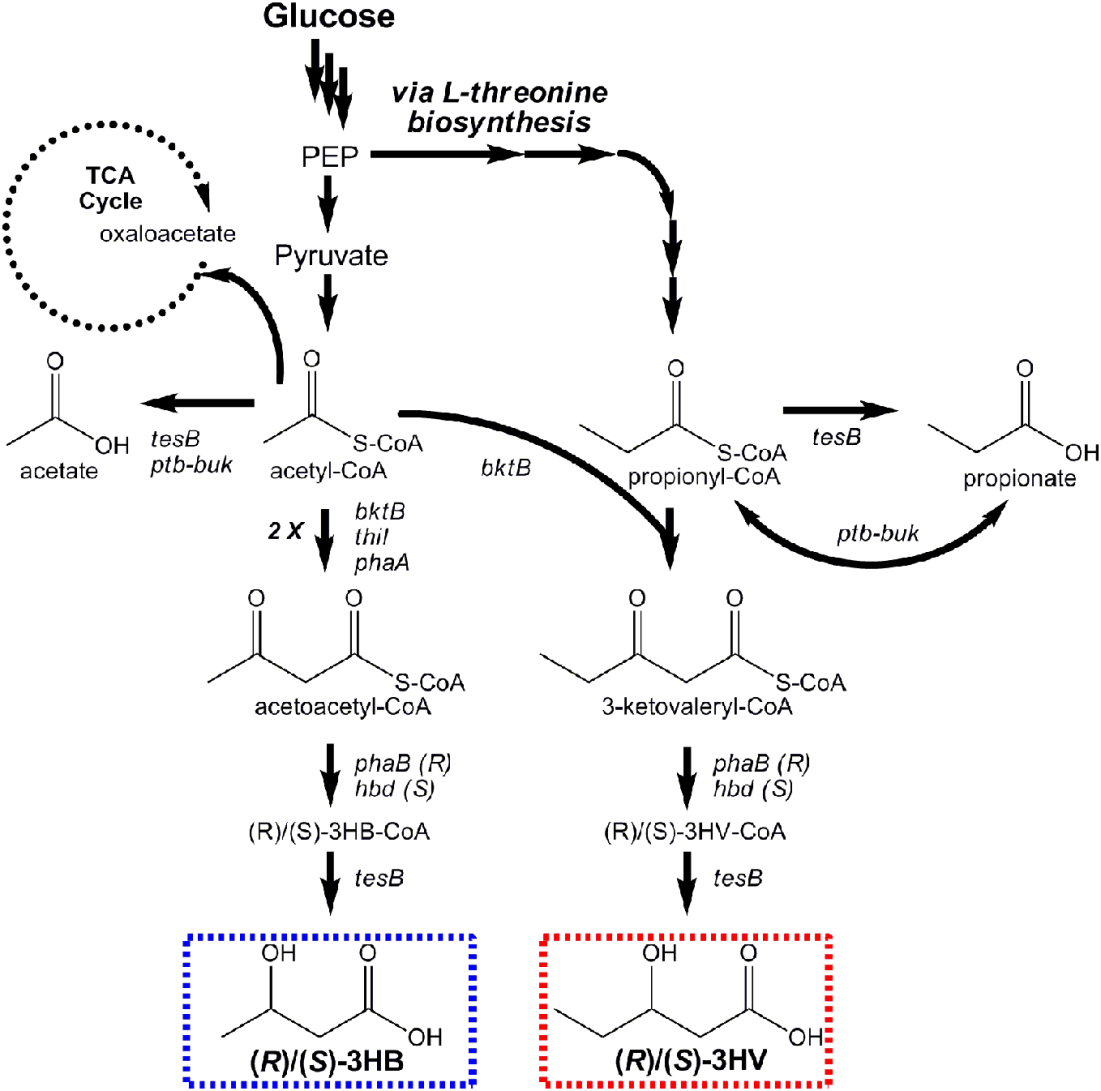
**Pathways engineered for stereoselective production of *(R)*- and *(S)*-3-hydroxybutyrate and *(R)*- and *(S)*-3-hydroxyvalerate from glucose**.

Microbial production of *(R)*-3HB by recombinant *E. coli* was first reported by Gao et al. Their engineered pathway was composed of *phaA* and *phaB* from *Ralstonia eutropha* (a natural PHB producer), encoding β-ketothiolase and an (*R*)-specific 3-hydroxybutyryl-CoA (*R*-3HB-CoA) dehydrogenase, respectively, to convert endogenous acetyl-CoA to 3-HB-CoA through acetoacetyl-CoA (Figure 6). The *ptb-buk* operon from *Clostridium acetobutylicum* was lastly co-expressed to convert *R*-3-HB-CoA to *R*-3HB (via *R*-3-hydroxybutyryl phosphate), and shake flask titers exceeding 2 g/L resulted (Gao et al., 2002). Tseng et al. later built upon these works, adding the ability to produce both (*R*)- and (*S*)-3HB isomers with high stereoselectivity and increasing product titers (Tseng et al., 2009). Stereoselective control was achieved by utilizing two different enantioselective 3HB-CoA dehydrogenases: (*R*)-selective PhaB from *R. eutropha* and (*S*)-selective Hbd from *Clostridium acetobutylicum*. It was also demonstrated that the broad-substrate thioesterase II of *E. coli* (encoded by *tesB*) effectively converted both *(R)*- and *(S)*-3HB-CoA to their respective 3HB isomer products, whereas *ptb-buk* was (*R*)-specific (Tseng et al., 2009). In the end, (*R*)- and (*S*)-3HB titers as high as 2.92 and 2.08 g/L, respectively, were attained. (*R*)-3HB biomonomer, can be used to make biodegradable PHB with well-controlled properties. (*S*)-3HB biomonomer, meanwhile, can be used to produce novel PHBs with new features and properties.

Prather and coworkers subsequently expanded upon their efforts to produce, for the first time, enantiomerically pure stereoisomers of the 5-carbon β-hydroxyacid 3HV (Tseng et al., 2010). This was achieved using a single renewable substrate (glucose) by first engineering *E. coli* to over-produce precursor propionyl-CoA via manipulation of the native L-threonine biosynthesis pathway. Propionyl-CoA was condensed with acetyl-CoA via a thiolase displaying broad substrate specificity (BktB of *R. eutropha*). The intermediate product, 3-ketovaleryl-CoA, is the 5-carbon analog to acetoacetyl-CoA in the 3HB pathway (Figure 6). Two analogous steps were then used for its stereoselective reduction to (*R*)- or (*S*)-3HV-CoA and conversion to the respective hydroxyacids. The use of BktB was essential in demonstrating the ability of biomonomer chain elongation at the initial condensation step. One disadvantage to their approach, however, resulted from the promiscuity of BktB, which led to the simultaneous production of acetoacetyl-CoA, and hence 3HB. This realization implies that control over product purity had diminished. Nevertheless, in glucose fed cultures, titers of 0.50 and 0.31 g/L of *(R)*- and *(S)*-3HV, respectively, were demonstrated. The subsequent discovery that NADP(H) limitations of PhaB reduced conversion to *(R)*-3HV was later overcome using glycerol as substrate, which improved final titers to as high as 0.96 g/L. Whereas 3HV production has yet be commercially explored, as a biomonomer it offers the potential develop bioplastics with broader ranges of properties.

## Diols

Short-chain diols suitable for bioplastics applications have been produced microbially via both natural and engineered pathways. For example, the four-carbon diol 2,3-butanediol (2,3-BDO) is a natural fermentation product of many microbes, including many species of *Klebsiella*, *Bacillus*, and lactic acid bacteria (Voloch et al., 1985). Microbial production of 2,3-BDO by both natural and engineered strains will not be discussed here, however, as it has been extensively reviewed over the years (Garg and Jain, 1995; Celinska and Grajek, 2009; Ji et al., 2010; Nielsen et al., 2010; Ji et al., 2011). Today, companies including LanzaTech (New Zealand) are developing 2,3-BDO as a biomonomer platform following its chemocatalytic conversion to butadiene. More recent efforts have focused on engineering microbes to produce the three-carbon diol 1,3-propanediol (1,3-PDO) and the four-carbon diol 1,4-butanediol (1,4-BDO). Both 1,3-PDO and 1,4-BDO are non-natural metabolites, produced only with the aid of novel, engineered metabolic pathways. Like 2,3-BDO, 1,4-BDO can be similarly converted to butadiene. More importantly, however, both 1,3-PDO and 1,4-BDO are directly useful as biomonomers for the polyesters poly(propylene terephthalate) (PPT) and poly(butylene terephthalate) (PBT). Both PPT and PBT have the potential to steal market share from what is perhaps the oldest polyester, polyethylene terephthalate (PET) (Haas et al., 2005).

**Figure 7:**
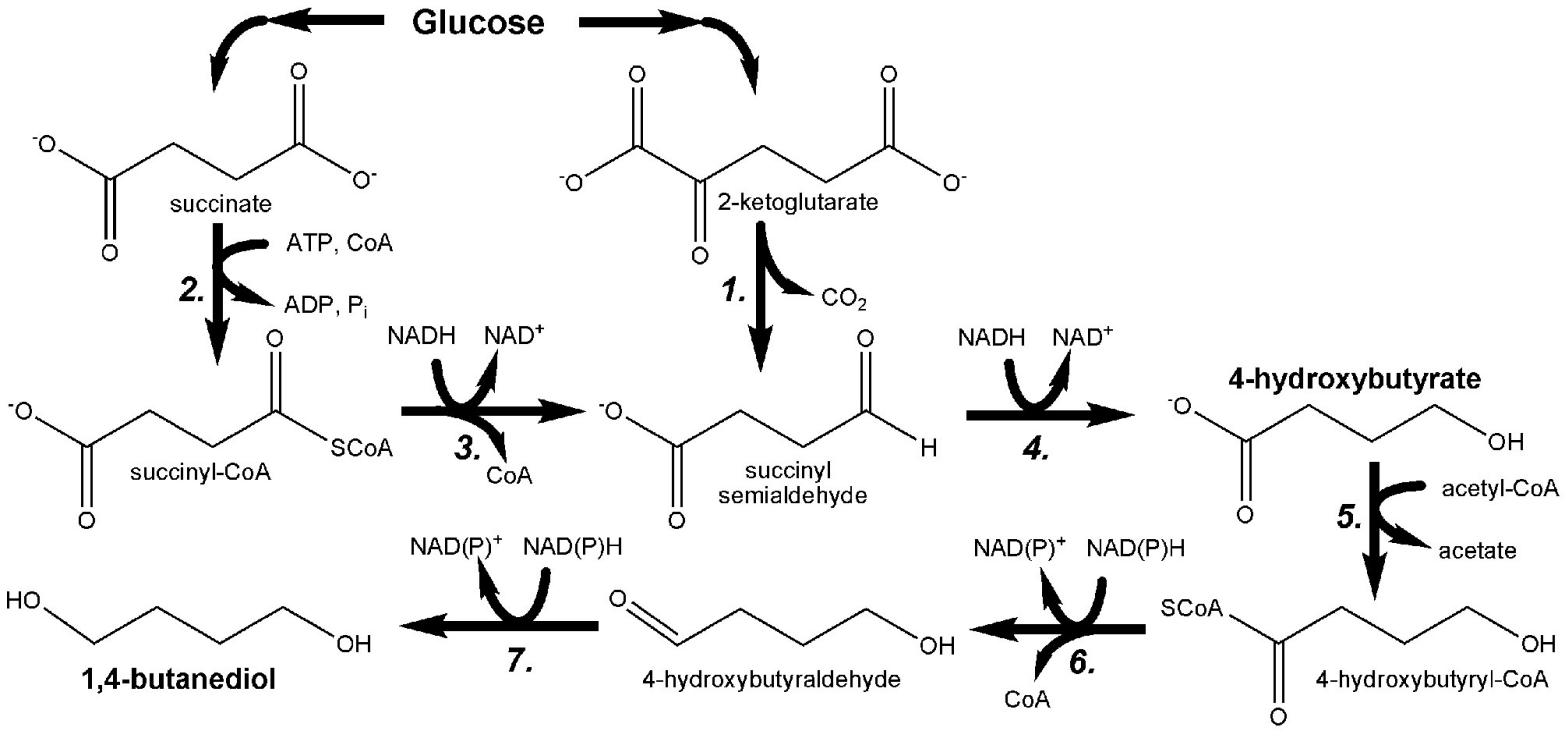
**Pathway engineered by Yim et al. for the microbial production of 1,4-butanediol (1,4-BDO) through the hydroxyacid intermediate 4-hydroxybutyrate (4HB)**. *1*. 2-oxoglutarate decarboxylase; *2*. succinyl-CoA synthetase; *3*. CoA-dependent succinate semialdehyde dehydrogenase; *4*. 4-hydroxybutyrate dehydrogenase; *5*. 4-hydroxybutyryl-CoA transferase; *6*. 4-hydroxybutyryl-CoA reductase; *7*. alcohol dehydrogenase.

While microbial production of 1,3-PDO has been recently and comprehensively reviewed (Nakamura and Whited, 2003; Sauer et al., 2008; Saxena et al., 2009; Celinska, 2010), the engineering of *E. coli* to produce up to 18 g/L 1,4-BDO from glucose (as well as other substrates) was only recently reported for the first time (Yim et al., 2011). Bioproduction of 1,4-BDO was accomplished through the development of two novel pathways (Figure 7), the design of which was facilitated using the SimPheny Biopathway Predictor; a design algorithm that considers chemical structure to predict possible routes from central metabolite precursors to end-product targets (Smolke, 2009). It is also noted that, as seen in Figure 7, both 1,4-BDO pathways include the common intermediate 4-hydroxybutyrate (4HB), a hydroxyacid. As described above, hydroxyacids are excellent precursors for polyester production. Thus, by further leveraging this technology an additional, non-natural hydroxyacid building block could also be added to the list of potential polyester biomonomers. As their flagship molecule, Genomatica (San Diego, CA) is now developing 1,4-BDO at the commercial scale. Moreover, NatureWorks and BioAmber recently formed a joint venture (AmberWorks) to explore the development of 100% renewable polyester co-polymers of 1,4-BDO and succinic acid (PBS).

## Conclusions

Whereas most monomer compounds and their respective polymers are presently derived from non-renewable petroleum, by application of metabolic engineering to create novel enzyme pathways this is beginning to change. The rapidly expanding number and diversity of biomonomers will continue to support the development of new markets for “green” and sustainable bioplastics with properties identical to their petroleum-derived counterparts. With the aid of continued research and development, renewable biomonomers will ultimately help to reduce global oil consumption while also promoting greater environmental sustainability.

### Conflict of interest statement

The authors declare that the research was conducted in the absence of any commercial or financial relationships that could be construed as a potential conflict of interest.

## References

[B1] Asada Y. Miyake M. Miyake J. Kurane R. Tokiwa Y. (1999). Photosynthetic accumulation of poly-(hydroxybutyrate) by cyanobacteria—the metabolism and potential for CO_2_ recycling. Int. J. Biol. Macromol. 25, 37–42 10.1016/S0141-8130(99)00013-6 10416648

[B2] Atsumi S. Liao J. C. (2008). Metabolic engineering for advanced biofuels production from *Escherichia coli*. Curr. Opin. Biotechnol. 19, 414–419 10.1016/j.copbio.2008.08.008 18761088 PMC2673505

[B3] Atsumi S. Hanai T. Liao J. C. (2008). Non-fermentative pathways for synthesis of branched-chain higher alcohols as biofuels. Nature 451, 86–89 10.1038/nature06450 18172501

[B4] Bechthold I. Bretz K. Kabasci S. Kopitzky R. Springer A. (2008). Succinic acid: a new platform chemical for biobased polymers from renewable resources. Chem. Eng. Technol. 31, 647–654

[B5] Bermudez M. Leon S. Aleman C. Munoz-Guerra S. (2000). Comparison of lamellar crystal structure and morphology of nylon 46 and nylon 5. Polymer 41, 8961–8973

[B6] Braunegg G. Lefebvre G. Genser K. F. (1998). Polyhydroxyalkanoates, biopolyesters from renewable resources: physiological and engineering aspects. J. Biotechnol. 65, 127–161 9828458 10.1016/s0168-1656(98)00126-6

[B7] Campbell C. D. Vederas J. C. (2010). Biosynthesis of lovastatin and related metabolites formed by fungal iterative PKS enzymes. Biopolymers 93, 755–763 10.1002/bip.21428 20577995

[B8] Carothers J. M. Goler J. A. Keasling J. D. (2009). Chemical synthesis using synthetic biology. Curr. Opin. Biotechnol. 20, 498–503 10.1016/j.copbio.2009.08.001 19720519

[B9] Celinska E. (2010). Debottlenecking the 1, 3-propanediol pathway by metabolic engineering. Biotechnol. Adv. 28, 519–530 10.1016/j.biotechadv.2010.03.003 20362657

[B10] Celinska E. Grajek W. (2009). Biotechnological production of 2, 3-butanediol-Current state and prospects. Biotechnol. Adv. 27, 715–725 10.1016/j.biotechadv.2009.05.002 19442714

[B11] Chen G. Q. (2009). A microbial polyhydroxyalkanoates (PHA) based bio- and materials industry. Chem. Soc. Rev. 38, 2434–2446 10.1039/b812677c 19623359

[B12] Curran K. A. Alper H. S. (2012). Expanding the chemical palate of cells by combining systems biology and metabolic engineering. Metab. Eng. 14, 289–297 10.1016/j.ymben.2012.04.006 22595280

[B13] Czaja W. Krystynowicz A. Bielecki S. Brown R. M. (2006). Microbial cellulose—the natural power to heal wounds. Biomaterials 27, 145–151 10.1016/j.biomaterials.2005.07.035 16099034

[B14] Erickson B. Nelson, Winters P. (2011). Perspective on opportunities in industrial biotechnology in renewable chemicals. Biotechnol. J. 7, 176–185 10.1002/biot.201100069 21932250 PMC3490365

[B15] Freedonia Group. (2009). World Bioplastics to 2013. Cleveland, OH: Freedonia

[B16] Gao H. J. Wu Q. Chen G. Q. (2002). Enhanced production of D-(-)-3-hydroxybutyric acid by recombinant *Escherichia coli*. FEMS Microbiol. Lett. 213, 59–65 12127489 10.1111/j.1574-6968.2002.tb11286.x

[B17] Garg S. K. Jain A. (1995). Fermentative production of 2, 3-butanediol – a review. Bioresour. Technol. 51, 103–109

[B18] Global Industry Analysts. (2012). Plastics: A Global Outlook. San Jose, CA: GIA

[B19] Haas T. Jaeger B. Weber R. Mitchell S. F. King C. F. (2005). New diol processes: 1, 3-propanediol and 1, 4-butanediol. Appl. Catal. A Gen. 280, 83–88

[B20] Hoenich N. (2006). Cellulose for medical applications: past, present, and future. Bioresources 1, 270–280

[B21] Hu Y. C. Zhan N. N. Dou C. Huang H. Han Y. W. Yu D. H. Hu Y. (2010). Selective dehydration of bio-ethanol to ethylene catalyzed by lanthanum-phosphorous-modified HZSM-5, influence of the fusel. Biotechnol. J. 5, 1186–1191 10.1002/biot.201000139 21058319

[B22] Igarashi K. Kashiwagi K. (2000). Polyamines: mysterious modulators of cellular functions. Biochem. Biophys. Res. Commun. 271, 559–564 10.1006/bbrc.2000.2601 10814501

[B23] Jiang X. Meng X. Xian M. (2009). Biosynthetic pathways for 3-hydroxypropionic acid production. Appl. Microbiol. Biotechnol. 82, 995–1003 10.1007/s00253-009-1898-7 19221732

[B24] Ji X. J. Huang H. Ouyang P. K. (2011). Microbial 2, 3-butanediol production: a state-of-the-art review. Biotechnol. Adv. 29, 351–364 10.1016/j.biotechadv.2011.01.007 21272631

[B25] Ji X. J. Nie Z. K. Li Z. Y. Gao Z. Huang H. (2010). Biotechnological production of 2, 3-butanediol. Prog. Chem. 22, 2450–2461

[B26] Jung Y. K. Kim T. Y. Park S. J. Lee S. Y. (2010). Metabolic engineering of *Escherichia coli* for the production of polylactic acid and its copolymers. Biotechnol. Bioeng. 105, 161–171 10.1002/bit.22548 19937727

[B27] Keshavarz T. Roy I. (2010). Polyhydroxyalkanoates: bioplastics with a green agenda. Curr. Opin. Microbiol. 13, 321–326 10.1016/j.mib.2010.02.006 20227907

[B28] Kind S. Jeong W. K. Schroder H. Wittmann C. (2010a). Systems-wide metabolic pathway engineering in *Corynebacterium glutamicum* for bio-based production of diaminopentane. Metab. Eng. 12, 341–351 10.1016/j.ymben.2010.03.005 20381632

[B29] Kind S. Jeong W. K. Schroder H. Zelder O. Wittmann C. (2010b). Identification and elimination of the competing N-acetyldiaminopentane pathway for improved production of diaminopentane by *Corynebacterium glutamicum*. Appl. Environ. Microbiol. 76, 5175–5180 10.1128/AEM.00834-10 20562290 PMC2916474

[B30] Kind S. Kreye S. Wittmann C. (2011). Metabolic engineering of cellular transport for overproduction of the platform chemical 1, 5-diaminopentane in *Corynebacterium glutamicum*. Metab. Eng. 13, 617–627 10.1016/j.ymben.2011.07.006 21821142

[B31] Kind S. Wittmann C. (2011). Bio-based production of the platform chemical 1, 5-diaminopentane. Appl. Microbiol. Biotechnol. 91, 1287–1296 10.1007/s00253-011-3457-2 21761208

[B32] Kosakai Y. Soo Park Y. Okabe M. (1997). Enhancement of L+-lactic acid production using mycelial flocs of Rhizopus oryzae. Biotechnol. Bioeng. 55, 461–470 10.1002/(SICI)1097-0290(19970805)55:3<461::AID-BIT1>3.0.CO;2-A 18636511

[B33] Krystynowicz A. Czaja W. Wiktorowska-Jezierska A. Goncalves-Miskiewicz M. Turkiewicz M. Bielecki S. (2002). Factors affecting the yield and properties of bacterial cellulose. J. Ind. Microbiol. Biotechnol. 29, 189–195 10.1038/sj.jim.7000303 12355318

[B34] Lee J. W. Kim H. U. Choi S. Yi J. Lee S. Y. (2011a). Microbial production of building block chemicals and polymers. Curr. Opin. Biotechnol. 22, 758–767 10.1016/j.copbio.2011.02.011 21420291

[B35] Lee J. W. Kim T. Y. Jang Y. S. Choi S. Lee S. Y. (2011b). Systems metabolic engineering for chemicals and materials. Trends Biotechnol. 29, 370–378 10.1016/j.tibtech.2011.04.001 21561673

[B36] Lee J. Na D. Park J. Lee J. Choi S. Lee S. (2012). Systems metabolic engineering of microorganisms for natural and non-natural chemical. Nat. Chem. Biol. 8, 536–546 10.1038/nchembio.970 22596205

[B37] Lee S. Y. Choi J. I. Wong H. H. (1999). Recent advances in polyhydroxyalkanoate production by bacterial fermentation: mini-review. Int. J. Biol. Macromol. 25, 31–36 10416647 10.1016/s0141-8130(99)00012-4

[B38] Le Vo T. Kim T. Hong S. (2012). Effects of glutamate decarboxylase and gamma-aminobutyric acid (GABA) transporter on the bioconversion of GABA in engineered *Escherichia coli*. Bioprocess Biosyst. Eng. 35, 645–650 10.1007/s00449-011-0634-8 21971608

[B39] Leonard E. Runguphan W. O'Connor S. Prather K. J. (2009). Opportunities in metabolic engineering to facilitate scalable alkaloid production. Nat. Chem. Biol. 5, 292–300 10.1038/nchembio.160 19377455

[B40] Lunt J. (1998). Large-scale production, properties and commercial applications of polylactic acid polymers. Polym. Degrad. Stabil. 59, 145–152

[B41] Martens J. H. Barg H. Warren M. J. Jahn D. (2002). Microbial production of vitamin B-12. Appl. Microbiol. Biotechnol. 58, 275–285 10.1007/s00253-001-0902-7 11935176

[B42] Martin C. H. Nielsen D. R. Solomon K. V. Prather K. L. J. (2009). Synthetic metabolism: engineering biology at the protein and pathway scales. Chem. Biol. 16, 277–286 10.1016/j.chembiol.2009.01.010 19318209

[B43] Mazumdar S. Clomburg J. M. Gonzalez R. (2010). *Escherichia coli* strains engineered for homofermentative production of D-lactic acid from glycerol. Appl. Environ. Microbiol. 76, 4327–4336 10.1128/AEM.00664-10 20472739 PMC2897450

[B44] McKenna R. Nielsen D. R. (2011). Styrene biosynthesis from glucose by engineered *E. coli*. Metab. Eng. 13, 544–554 10.1016/j.ymben.2011.06.005 21722749

[B45] Mendez-Perez D. Begemann M. B. Pfleger B. F. (2011). Modular synthase-encoding gene involved in alpha-olefin biosynthesis in *Synechococcus sp*. strain PCC 7002. Appl. Environ. Microbiol. 77, 4264–4267 10.1128/AEM.00467-11 21531827 PMC3131656

[B46] Mimitsuka T. Sawai H. Hatsu M. Yamada K. (2007). Metabolic engineering of *Corynebacterium glutamicum* for cadaverine fermentation. Biosci. Biotechnol. Biochem. 71, 2130–2135 17895539 10.1271/bbb.60699

[B47] Nakamura C. E. Whited G. M. (2003). Metabolic engineering for the microbial production of 1, 3-propanediol. Curr. Opin. Biotechnol. 14, 454–459 10.1016/j.copbio.2003.08.005 14580573

[B48] Nexant Chem Systems. (2009). New Process Evaluation and Research Program: Nylon 6 and Nylon 6, 6. San Francisco, CA: Nexant

[B49] Nielsen D. R. Yoon S. H. Yuan C. J. Prather K. L. (2010). Metabolic engineering of acetoin and meso-2, 3-butanediol biosynthesis in *E. coli*. Biotechnol. J. 5, 274–284 10.1002/biot.200900279 20213636

[B50] Niu W. Draths K. M. Frost J. W. (2002). Benzene-free synthesis of adipic acid. Biotechnol. Prog. 18, 201–211 10.1021/bp010179x 11934286

[B51] Pawlowski G. Dammel R. Eckes C. Lindley C. R. Meier W. Przybilla K. J. Roschert H. Spiess W. (1991). Substituted polyhydroxystyrenes as matrix resins for chemically amplified deep uv resist materials. Microelectron. Eng. 13, 29–32

[B52] Pei L. Schmidt M. Wei W. (2011). Conversion of biomass into bioplastics and their potential environmental impacts, in Biotechnology of Biopolymers, ed Elnashar M. (Rijeka, Croatia: InTech), 57–74

[B53] Peralta-Yahya P. P. Keasling J. D. (2010). Advanced biofuel production in microbes. Biotechnol. J. 5, 147–162 10.1002/biot.200900220 20084640

[B54] Phillips A. L. (2008). Bioplastics boom. Am. Sci. 96, 109

[B55] Plastics Europe. (2011). Plastics Europe Market Research Group, Plastics—The Facts 2011. Brussels, Belgium: Plastics Europe

[B56] Qian Z. G. Xia X. X. Lee S. Y. (2009). Metabolic engineering of *Escherichia coli* for the production of putrescine: a four carbon diamine. Biotechnol. Bioeng. 104, 651–662 10.1002/bit.22502 19714672

[B58] Qian Z. G. Xia X. X. Lee S. Y. (2011). Metabolic engineering of *Escherichia coli* for the production of cadaverine: a five carbon diamine. Biotechnol. Bioeng. 108, 93–103 10.1002/bit.22918 20812259

[B60] Qi W. W. Vannelli T. Breinig S. Ben-Bassat A. Gatenby A. A. Haynie S. L. Sariaslani F. S. (2007). Functional expression of prokaryotic and eukaryotic genes in *Escherichia coli* for conversion of glucose to p-hydroxystyrene. Metab. Eng. 9, 268–276 10.1016/j.ymben.2007.01.002 17451990

[B61] Rathnasingh C. Raj S. M. Jo J. E. Park S. (2009). Development and evaluation of efficient recombinant *Escherichia coli* strains for the production of 3-hydroxypropionic acid from glycerol. Biotechnol. Bioeng. 104, 729–739 10.1002/bit.22429 19575416

[B62] Rathnasingh C. Raj S. M. Lee Y. Catherine C. Ashok S. Park S. (2012). Production of 3-hydroxypropionic acid via malonyl-CoA pathway using recombinant *Escherichia coli* strains. J. Biotechnol. 157, 633–640 10.1016/j.jbiotec.2011.06.008 21723339

[B63] Reddy G. Altaf M. Naveena B. J. Venkateshwar M. Kumar E. V. (2008). Amylolytic bacterial lactic acid fermentation–a review. Biotechnol. Adv. 26, 22–34 10.1016/j.biotechadv.2007.07.004 17884326

[B64] Reinecke F. Steinbuchel A. (2009). Ralstonia eutropha strain H16 as model organism for PHA metabolism and for biotechnological production of technically interesting biopolymers. J. Mol. Microbiol. Biotechnol. 16, 91–108 10.1159/000142897 18957865

[B65] Revelles O. Wittich R.-M. Ramos J. L. (2007). Identification of the initial steps in d-lysine catabolism in *Pseudomonas putida*. J. Bacteriol. 189, 2787–2792 10.1128/JB.01538-06 17259313 PMC1855791

[B66] Ro D. K. Paradise E. M. Ouellet M. Fisher K. J. Newman K. L. Ndungu J. M. Ho K. A. Eachus R. A. Ham T. S. Kirby J. Chang M. C. Withers S. T. Shiba Y. Sarpong R. Keasling J. D. (2006). Production of the antimalarial drug precursor artemisinic acid in engineered yeast. Nature 440, 940–943 10.1073/pnas.1110740109 16612385

[B67] Rojas-Rosas O. Villafana-Rojas J. Lopez-Dellamary F. A. Nungaray-Arellano J. Gonzalez-Reynoso O. (2007). Production and characterization of polyhydroxyalkanoates in *Pseudomonas aeruginosa* ATCC (9027). from glucose, an unrelated carbon source. Can. J. Microbiol. 53, 840–851 10.1139/W07-023 17898839

[B68] Ross P. Mayer R. Benziman M. (1991). Cellulose biosynthesis and function in bacteria. Microbiol. Rev. 55, 35–58 2030672 10.1128/mr.55.1.35-58.1991 PMC372800

[B69] Rude M. A. Baron T. S. Brubaker S. Alibhai M. Del Cardayre S. B. Schirmer A. (2011). Terminal olefin (1-alkene) biosynthesis by a novel p450 fatty acid decarboxylase from *Jeotgalicoccus* species. Appl. Environ. Microbiol. 77, 1718–1727 10.1128/AEM.02580-10 21216900 PMC3067255

[B70] Sauer M. Marx H. Mattanovich D. (2008). Microbial production of 1, 3-propanediol. Recent Pat. Biotechnol. 2, 191–197 19075867 10.2174/187220808786240999

[B71] Saxena R. K. Anand P. Saran S. Isar J. (2009). Microbial production of 1, 3-propanediol: recent developments and emerging opportunities. Biotechnol. Adv. 27, 895–913 10.1016/j.biotechadv.2009.07.003 19664701

[B72] Schirmer A. Rude M. A. Li X. Popova E. Del Cardayre S. B. (2010). Microbial biosynthesis of alkanes. Science 329, 559–562 10.1126/science.1187936 20671186

[B73] Schneider J. Wendisch V. (2010). Putrescine production by engineered *Corynebacterium glutamicum*. Appl. Microbiol. Biotechnol. 88, 859–868 10.1007/s00253-010-2778-x 20661733

[B74] Schubert P. Steinbuchel A. Schlegel H. G. (1988). Cloning of the *Alcaligenes eutrophus* genes for synthesis of poly-beta-hydroxybutyric acid (PHB) and synthesis of PHB in *Escherichia coli*. J. Bacteriol. 170, 5837–5847 2848014 10.1128/jb.170.12.5837-5847.1988 PMC211690

[B75] Singh M. Patel S. K. S. Kalia V. C. (2009). *Bacillus subtilis* as potential producer for polyhydroxyalkanoates. Microbial. Cell Fact. 8, 38–48 10.1186/1475-2859-8-38 19619289 PMC2719590

[B76] Smolke C. D. (2009). The Metabolic Pathway Engineering Handbook Fundamentals. Boca Raton, FL: CRC Press/Taylor and Francis

[B77] Sodergard A. Stolt M. (2002). Properties of lactic acid based polymers and their correlation with composition. Prog. Polym. Sci. 27, 1123–1163

[B78] Song H. Lee S. Y. (2006). Production of succinic acid by bacterial fermentation. Enzyme Microb. Technol. 39, 352–361

[B79] Sri. (2010). Styrene. Access Intelligence LLC Inc 10.1111/j.1750-3841.2009.01206.x 19723212

[B80] Steen E. J. Kang Y. Bokinsky G. Hu Z. Schirmer A. Mcclure A. Del Cardayre S. B. Keasling J. D. (2010). Microbial production of fatty-acid-derived fuels and chemicals from plant biomass. Nature 463, 559–562 10.1038/nature08721 20111002

[B81] Straathof A. J. J. Sie S. Franco T. T. Van Der Wielen L. A. M. (2005). Feasibility of acrylic acid production by fermentation. Appl. Microbiol. Biotechnol. 67, 727–734 10.1007/s00253-005-1942-1 15735954

[B82] Suthers P. F. Cameron D. C. (2000). Production of 3-hydroxypropionic acid in recombinant organisms. US Patent 6852517.

[B83] Tabor C. W. Tabor H. (1985). Polyamines in microorganisms. Microbiol. Rev. 49, 81–99 3157043 10.1128/mr.49.1.81-99.1985 PMC373019

[B84] Takahara I. Saito M. Inaba M. Murata K. (2005). Dehydration of ethanol into ethylene over solid acid catalysts. Catal. Lett. 105, 249–252

[B85] Tateno T. Okada Y. Tsuchidate T. Tanaka T. Fukuda H. Kondo A. (2009). Direct production of cadaverine from soluble starch using *Corynebacterium glutamicum* coexpressing alpha-amylase and lysine decarboxylase. Appl. Microbiol. Biotechnol. 82, 115–121 10.1007/s00253-008-1751-4 18989633

[B86] Thakker C. Martinez I. San K. Y. Bennett G. N. (2012). Succinate production in *Escherichia coli*. Biotechnol. J. 7, 213–224 10.1002/biot.201100061 21932253 PMC3517001

[B87] Thompson R. C. Swan S. H. Moore C. J. Vom Saal F. S. (2009). Our plastic age. Philos. Trans. R. Soc. B Biol. Sci. 364, 1973–1976 10.1098/rstb.2009.0054 19528049 PMC2874019

[B88] Thorball V. (1967). Styrofoam—an extruded polystyrene foam for low temperature insulation. Mod. Refrig. Air Cond. 70, 51

[B89] Tseng H. C. Harwell C. L. Martin C. H. Prather K. L. (2010). Biosynthesis of chiral 3-hydroxyvalerate from single propionate-unrelated carbon sources in metabolically engineered *E. coli*. Microb. Cell Fact. 9, 96 10.1186/1475-2859-9-96 21110891 PMC3000843

[B90] Tseng H. C. Martin C. H. Nielsen D. R. Prather K. L. (2009). Metabolic engineering of *Escherichia coli* for enhanced production of (R)- and (S)-3-hydroxybutyrate. Appl. Environ. Microbiol. 75, 3137–3145 10.1128/AEM.02667-08 19304817 PMC2681625

[B91] Tsuruta H. Paddon C. J. Eng D. Lenihan J. R. Horning T. Anthony L. C. Regentin R. Keasling J. D. Renninger N. S. Newman J. D. (2009). High-level production of amorpha-4, 11-diene, a precursor of the antimalarial agent artemisinin, in *Escherichia coli*. PLoS ONE 4:e4489 10.1371/journal.pone.0004489 19221601 PMC2637983

[B92] US Department of Energy. (2002). Steam System Opportunity Assessment for the Pulp and Paper, Chemical Manufacturing, and Petroleum Refining Industries. Washington, DC

[B93] US Department of Energy. (2004). Top Value Added Chemicals from Biomass. Washington, DC

[B94] US Energy Information Administration. (2011). Available online at www.eia.gov

[B95] Voloch M. Jjansen N. B. Ladisch M. Tsao G. T. Narayan R. Rodwell V. W. (1985). 2, 3-Butanediol, in Comprehensive Biotechnology: The Principles, Applications and Regulations of Biotechnology in Industry, Agriculture and Medicine, 1st Edn, ed Moo-Young M. (New York, NY: Pergamon Press).

[B96] Wee Y. J. Kim J. N. Ryu H. W. (2006). Biotechnological production of lactic acid and its recent applications. Food Technol. Biotechnol. 44, 163–175

[B97] Whited G. Feher F. Benko D. Cervin M. Chotani G. Mcauliffe J. Laduca R. Ben-Shoshan E. Sanford K. (2010). Development of a gas-phase bioprocess for isoprene-monomer production using metabolic pathway engineering. Ind. Biotechnol. 6, 152–163

[B98] Wierckx N. J. Ballerstedt H. De Bont J. A. Wery J. (2005). Engineering of solvent-tolerant *Pseudomonas putida* S12 for bioproduction of phenol from glucose. Appl. Environ. Microbiol. 71, 8221–8227 10.1128/AEM.71.12.8221-8227.2005 16332806 PMC1317433

[B99] Wu C. Koylinski T. Bozik J. (1981). Preparation of styrene from ethylbenzene US Patent 4255599.

[B100] Xu J. Guo B. H. (2010). Poly(butylene succinate) and its copolymers: research, development and industrialization. Biotechnol. J. 5, 1149–1163 10.1002/biot.201000136 21058317

[B101] Yang T. H. Kim T. W. Kang H. O. Lee S. H. Lee E. J. Lim S. C. Oh S. O. Song A. J. Park S. J. Lee S. Y. (2010). Biosynthesis of polylactic acid and its copolymers using evolved propionate CoA transferase and PHA synthase. Biotechnol. Bioeng. 105, 150–160 10.1002/bit.22547 19937726

[B102] Yim H. Haselbeck R. Niu W. Pujol-Baxley C. Burgard A. Boldt J. Khandurina J. Trawick J. D. Osterhout R. E. Stephen R. Estadilla J. Teisan S. Schreyer H. B. Andrae S. Yang T. H. Lee S. Y. Burk M. J. Van Dien S. (2011). Metabolic engineering of *Escherichia coli* for direct production of 1, 4-butanediol. Nat. Chem. Biol. 7, 445–452 10.1038/nchembio.580 21602812

[B103] Zhou S. Causey T. B. Hasona A. Shanmugam K. T. Ingram L. O. (2003). Production of optically pure D-lactic acid in mineral salts medium by metabolically engineered *Escherichia coli* W3110. Appl. Environ. Microbiol. 69, 399–407 12514021 10.1128/AEM.69.1.399-407.2003 PMC152430

[B104] Zhou Y. Dominguez J. M. Cao N. Du J. Tsao G. T. (1999). Optimization of L-lactic acid production from glucose by *Rhizopus oryzae* ATCC 52311. Appl. Biochem. Biotechnol. 77–79, 401–407 15304710 10.1385/abab:78:1-3:401

